# The Flexibility of Ectopic Lipids

**DOI:** 10.3390/ijms17091554

**Published:** 2016-09-14

**Authors:** Hannah Loher, Roland Kreis, Chris Boesch, Emanuel Christ

**Affiliations:** 1 Division of Endocrinology, Diabetes and Clinical Nutrition, Inselspital, Bern University Hospital, University of Bern, CH-3010 Bern, Switzerland; hannah.loher@bluewin.ch; 2 Department of Clinical Research & Institute of Interventional, Diagnostic and Pediatric Radiology, University of Bern, CH-3010 Bern, Switzerland; roland.kreis@insel.ch (R.K.); chris.boesch@insel.ch (C.B.)

**Keywords:** ectopic lipids, insulin resistance, exercise, fasting, diabetes mellitus, growth hormone deficiency, athlete’s paradox, intramyocellular lipids, intrahepatocellular lipids, intracardiomyocellular lipids

## Abstract

In addition to the subcutaneous and the visceral fat tissue, lipids can also be stored in non-adipose tissue such as in hepatocytes (intrahepatocellular lipids; IHCL), skeletal (intramyocellular lipids; IMCL) or cardiac muscle cells (intracardiomyocellular lipids; ICCL). Ectopic lipids are flexible fuel stores that can be depleted by physical exercise and repleted by diet. They are related to obesity and insulin resistance. Quantification of IMCL was initially performed invasively, using muscle biopsies with biochemical and/or histological analysis. ^1^H-magnetic resonance spectroscopy (^1^H-MRS) is now a validated method that allows for not only quantifying IMCL non-invasively and repeatedly, but also assessing IHCL and ICCL. This review summarizes the current available knowledge on the flexibility of ectopic lipids. The available evidence suggests a complex interplay between quantitative and qualitative diet, fat availability (fat mass), insulin action, and physical exercise, all important factors that influence the flexibility of ectopic lipids. Furthermore, the time frame of the intervention on these parameters (short-term vs. long-term) appears to be critical. Consequently, standardization of physical activity and diet are critical when assessing ectopic lipids in predefined clinical situations.

## 1. Introduction

Obesity is related to the insulin resistance syndrome including type 2 diabetes mellitus, hypertension [1,2], non-alcoholic fatty liver disease (NAFLD) [3,4], and, consequently, increased risk for cardiovascular morbidity and mortality [5,6,7]. It has been established that besides the absolute amount of fat, its tissue-specific distribution plays a major role as a risk factor for cardiovascular disease [8].

Visceral obesity is well known to be associated with higher all-cause mortality [9]. It is also linked to increased risk for cardiovascular morbidity and mortality [10,11], as well as with type 2 diabetes [12,13,14]. Similarly, recent evidence suggests that the accumulation of epicardial adipose tissue around the heart is associated with coronary heart disease in humans [15].

In addition to the subcutaneous and the visceral fat tissue, lipids can also be stored in non-adipose tissues such as in hepatocytes (intrahepatocellular lipids; IHCL), skeletal (intramyocellular lipids; IMCL) or cardiac muscle cells (intracardiomyocellular lipids; ICCL), and pancreatic beta cells [16]. This fat is called ectopic fat [17]. Most importantly, the amount of visceral fat mass has been related to ectopic fat deposits [18,19,20], indicating an interaction between the different lipid deposits. This also implies that ectopic lipids are closely related to cardiovascular morbidity.

The pathophysiological link between ectopic lipids and cardiovascular morbidity lies in the impaired insulin action on target tissues (liver, muscle), which is influenced by ectopic lipid deposits. The first studies investigating these relations were published ca. 20 years ago and suggested that in particular the amounts of IMCL and IHCL are related to insulin resistance [21,22]. More recent data indicate that ectopic lipids can be influenced by diet [23,24,25,26,27,28,29,30,31,32,33,34,35,36,37,38,39,40,41,42] and physical exercise [32,37,38,43,44,45,46,47,48,49,50,51,52,53,54,55,56,57,58,59,60,61,62,63,64,65,66,67] (i.e., lifestyle intervention).

This review focuses on ectopic lipids, in particular on the flexibility of these lipid deposits whereby the term “flexibility” is used to describe changes in the amount of ectopic lipid content following a stimulus/intervention. Data investigating the flexibility of ectopic lipids in skeletal muscle have been extensively reported [37,38,43,44,45,46,47,48,49,50,51,52,53,54,55,65,66,67,68]. However, data on the impact of an acute bout of physical exercise on IHCL and ICCL are scarce [43,44,67,69,70] and not available with regard to pancreatic ectopic lipids. In addition, the underlying mechanisms of the flexibility of ectopic lipids are not completely understood.

Most of the available data regarding the flexibility of ectopic lipids in humans are based on healthy subjects (mainly males), such as sedentary lean and obese volunteers or endurance-trained athletes. Studies on the flexibility of ectopic lipids in patients are mainly limited to insulin resistant, i.e., glucose intolerant patients or patients with type 2 diabetes [71,72,73,74,75], but this data is mainly limited to long-term interventions. Some data exist in patients with type 1 diabetes or hypopituitarism [65,66,67].

The first studies that investigated the flexibility of ectopic lipids were performed using skeletal muscle biopsies before and after physical exercise [32,58,59,60,61,62,63,64,76,77,78,79]. Later, ^1^H-magnetic resonance spectroscopy (^1^H-MRS) became a reliable tool to assess IMCL as well as IHCL and ICCL non-invasively. Hence, repeated measurements of ectopic lipids became feasible.

This review summarizes the current knowledge on the flexibility of ectopic lipids (IMCL, IHCL ICCL) in humans. The main focus is on the influencing factors of ectopic lipids, namely physical exercise and diet.

This narrative review summarizes the current knowledge on the flexibility of ectopic lipids (IMCL, IHCL ICCL) in humans. The main focus is on the influencing factors of ectopic lipids, namely physical exercise and diet. PubMed was used and the search terms were intramyocellular lipids, skeletal muscle lipids, intrahepatic fat, intrahepatocellular lipids, intracardiomycellular lipids, intramuscular triglycerides, ectopic fat, ectopic lipids, exercise, fat, diet, lipid infusion, MR-spectroscopy, and bariatric surgery.

## 2. Methods to Assess Ectopic Lipids

Ectopic lipids in skeletal muscle have been quantified for decades using biochemical analysis of muscle tissue, which was extracted through biopsies mainly from M. vastus lateralis [80]. This method has been used for quantification of IMCL in physiological and clinical studies until today [59,81,82,83,84,85]. Even though it has been the most widely used method, biochemical analysis of muscle tissue is inaccurate. Three simultaneous muscle biopsies in the same muscle of the same subject showed a range of 24% in triacylglycerol content [81]. Although the visible fat had been removed before biochemical analysis, it is likely that extramuscular triacylglycerol was still present in many of the samples [81], resulting in less reliable results [81,86]. In addition, biopsies are based on an invasive method and repeated assessment is not always feasible. However, the investigation of biopsies allows for additional information such as biochemical pathways and structural analysis using EM [86] or histological analysis using oil red O staining [21,48,60,87].

In the 1990s, a non-invasive method was introduced to measure IMCL by means of ^1^H-MRS. It was first described by Schick et al. [88] and then validated and established by Boesch et al. [57,89,90,91]. Quantification of IMCL using ^1^H-MRS correlated well with EM analysis from biopsy samples, while biochemical analysis of biopsies was correlated neither with ^1^H-MRS nor with EM analyses [86]. The coefficient of variation of ^1^H-MRS for the assessment of IMCL is around 6% [57].

Liver fat is usually quantified using liver biopsies [92]. Obviously, because of its invasiveness, it cannot be performed repetitively in studies with healthy volunteers, yet it is still the gold standard for the diagnosis of NAFLD [92]. ^1^H-MRS is a good alternative because it is a non-invasive and non-ionizing procedure that allows for the estimation of hepatic fat and may be useful in follow-ups with patients with fatty liver disease [93,94,95,96]. Studies comparing the assessment of steatosis by ^1^H-MRS and histology showed a close correlation between the two methods [97,98]. When measured twice, IHCL levels were highly correlated (*r* = 0.99), pointing to good reproducibility [99]. For the determination of IHCL levels above those encountered in lean healthy subjects, MR imaging with various forms of the so-called Dixon technique is also available for repeated non-invasive determination of IHCL [100].

Ectopic lipids in cardiac muscle are less investigated; however, ^1^H-MRS also provides a reliable tool [101,102,103] to investigate this tissue. Validation of ^1^H-MRS in cardiac muscle with biopsy has been done during heart transplantation procedures. A biopsy of the myocardium and a ^1^H-MRS measurement before heart transplantation showed a high correlation (*r*^2^ = 0.83) of in vivo and ex vivo measurements [104]. In repeated measurements using respiratory navigator gating, the correlation coefficient of 0.81 indicates a good reproducibility of ^1^H-MRS in ICCL quantification [105].

An in-depth view and critical appraisal of the ^1^H-MRS method in assessing ectopic lipids has been covered by other reviews [90] and goes beyond the scope of this article. The current review focuses on the physiological factors that influence the flexibility of ectopic lipids.

**Figure 1:**
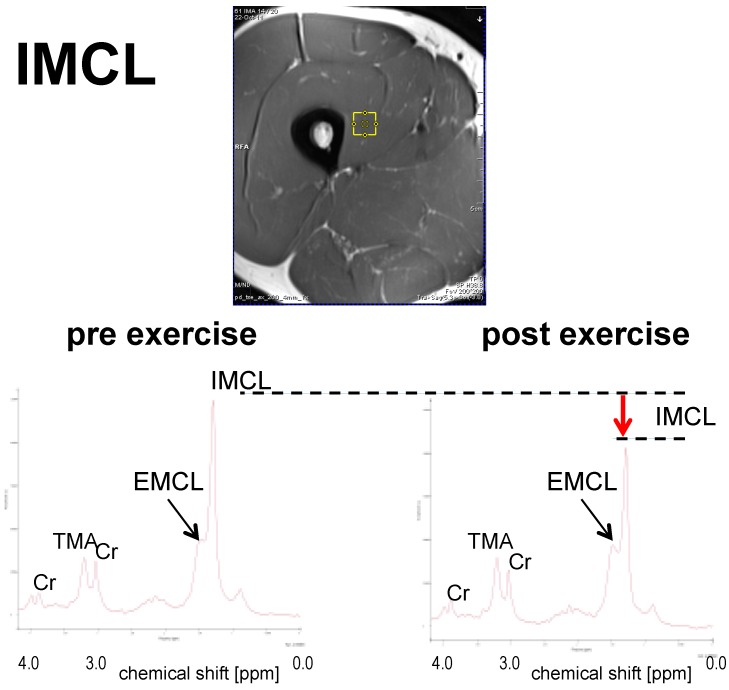
Sample ^1^H-MR spectra for the quantification of IMCL obtained from m. vastus intermedius before and after an exercise bout of 2 h: The largest peak in the spectrum originates from the aliphatic methylene groups in the fatty acid chains of IMCL. Direct comparison of the pre- and post-exercise spectra shows that IMCL were consumed in the exercise. Other peaks originate from further protons on the IMCL lipid chains, but also from the partially overlapping spectrum of extramyocellular lipids (EMCL, see e.g., [90] for details), as well as creatines (CH2 at 3.9 ppm and CH3 at 3.0 ppm) and trimethyl-ammonium (TMA) groups from metabolites, like carnitine, or the phosphocholines. (For details of the acquisition methods, see the electronic supplement to [43]; in short: single volume (~1.5 cm^3^), double spin echo localization, echo time 30 ms, 3T). The spin-echo image above the spectra shows the typical location where the spectra were obtained.

**Figure 2:**
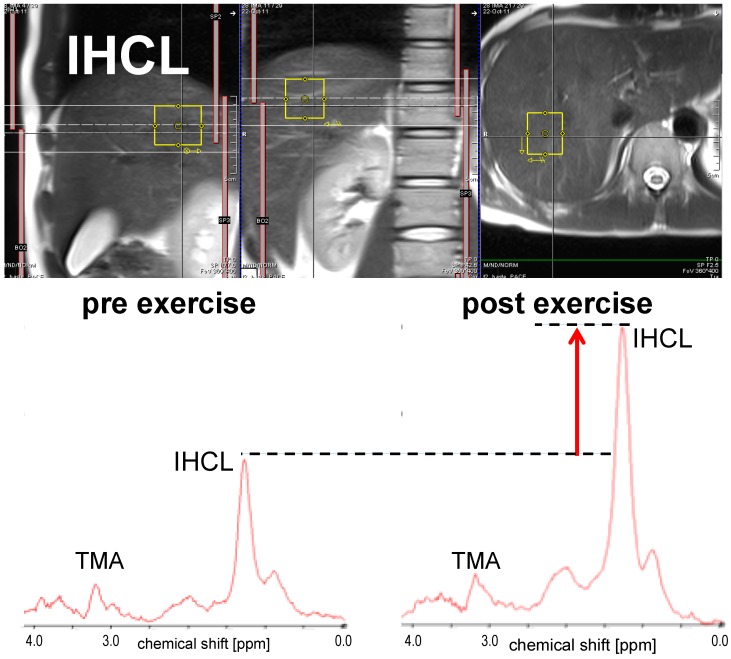
Sample ^1^H-MR spectra for the quantification of IHCL obtained before and after an exercise bout of 2 h: The largest peak in the spectrum originates from the aliphatic methylene groups in the fatty acid chains of IHCL. Direct comparison of the pre- and post-exercise spectra shows that IHCL were built up during/after the exercise. Other peaks originate from further protons on the IHCL lipid chains, and trimethyl-ammonium (TMA) groups from metabolites, like betain, or the phosphocholines (for details of the acquisition methods, see [43]; in short: single volume (~19 cm^3^), stimulated echo localization, echo time 13 ms, 3T, spectra obtained in sync with respiration, triggered for acquisition in expiration). The spin-echo images above the spectra that had also been obtained in expiration show the typical location where the spectra were obtained.

**Figure 3:**
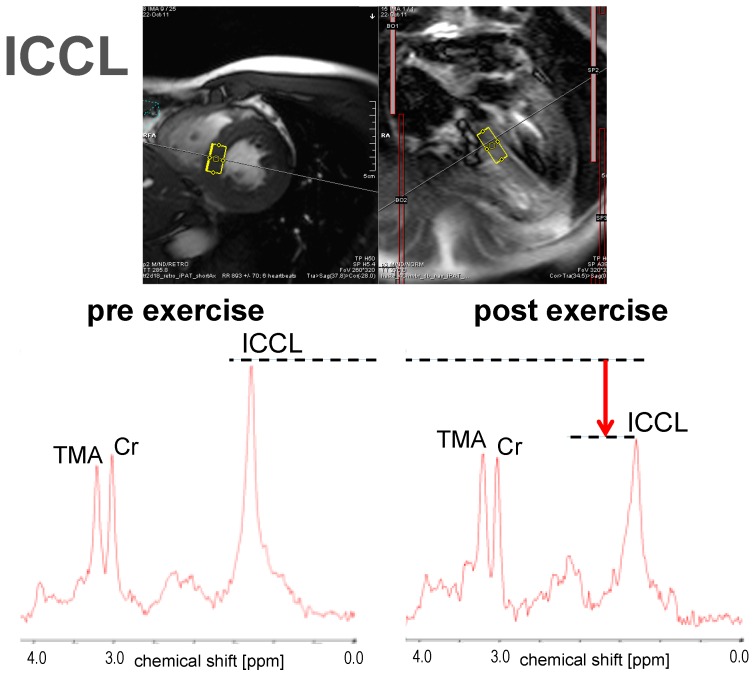
Sample ^1^H-MR spectra for the quantification of ICCL obtained before and after an exercise bout of 2 h: The largest peak in the pre-exercise spectrum originates from the aliphatic methylene groups in the fatty acid chains of ICCL. Direct comparison of the pre- and post-exercise spectra shows that ICCL were consumed during/after the exercise. Other peaks originate from further protons on the ICCL lipid chains, and creatines (CH_2_ at 3.9 ppm and CH_3_ at 3.0 ppm) and trimethyl-ammonium (TMA) groups from metabolites, like carnitine, or the phosphocholines. (For details of the acquisition methods, see [43]; in short: single volume (~5 cm^3^), double spin echo localization, echo time 35 ms, 3T, spectra obtained in sync with respiration and the cardiac cycle, triggered for acquisition in both expiration (based on realtime MR-images) and in end-systole (derived from the ECG signal)). The spin-echo images above the spectra that had also been obtained with double triggering and in the same respiratory and cardiac phase show the typical location where the spectra were obtained.

Examples for the measurement of ectopic lipids using ^1^H-MRS are shown in Figure 1, Figure 2 and Figure 3.

The examples were drawn from a recent study on the flexibility of ectopic lipids as a consequence of short-term exercise [43]. They represent spectra from skeletal muscle (vastus lateralis, Figure 1), the liver (Figure 2), and the heart (cardiac septum, Figure 3), obtained from single subjects before and immediately after an exercise bout. Dashed lines and arrows represent the changes in lipid levels graphically, while model-fitting evaluations must be used for quantitative measures (often with the use of the tissue water signal as a calibration standard). The presented examples were obtained with single volume MRS methods (for acquisition parameters see details in the figure legends), but other methodology that may give information from multiple locations simultaneously (see e.g., [106] for skeletal muscle, [107] for the liver, [108] for the heart) can be used as well.

## 3. The Effect of Physical Exercise on Ectopic Lipids

The effect of physical exercise on ectopic fat, especially on IMCL, has been evaluated in several clinical studies [32,37,38,39,43,44,45,46,47,48,49,50,51,52,53,54,55,56,58,59,60,61,62,63,64,65,66,67,68,71,72,73,74,75,76,77,78,79,83,84,85,109,110,111,112,113,114,115,116,117,118,119,120,121,122,123,124,125,126,127]. There is evidence for an acute “pre-post-exercise” as well as a long-term “training” effect of exercise on IMCL.

### 3.1. Short-Term Effect: Single Bout of Exercise

Consistently, ^1^H-MRS [37,38,43,44,45,46,47,48,49,50,51,52,53,54,55,56,57,65,66,67,68] and biopsy measurements [21,32,58,59,60,61,62,63,64,77,78,79] have shown that IMCL decrease after acute short-term exercise (45 min–3 h) at 50%–90% VO_2_ max in healthy subjects [32,43,44,45,46], endurance-trained athletes [21,37,38,46,47,48,49,50,51,52,53,54,55,56,57,58,59,60,61,62,63,64], patients with type 1 diabetes [65], and hypopituitary patients with growth hormone deficiency before and after growth hormone replacement therapy [66,67]. Not only aerobic endurance exercise but also resistance exercises decreased IMCL [78,127]. These results indicate that IMCL can be considered as a flexible fuel store that is depleted after physical exercise.

The particular interest in studying patients with hormone disorders lies in the fact that hormones regulate metabolism. Insulin is a strong inhibitor of lipolysis [128], whereas growth hormone increases lipolysis [129,130,131]. During physical exercise, insulin secretion decreases and growth hormone and catecholamine secretion increases, resulting in an increase in free fatty acids’ (FFA) concentration in the plasma. This is paralleled by an increase in fat availability within the working tissues such as skeletal muscle and heart muscle [43]. Patients with type 1 diabetes are an interesting model since glucose and insulin levels can be manipulated, thereby investigating carbohydrate, protein, and fat metabolism (locally and systemically) in the presence of high glucose and low insulin levels or euglycemia and high insulin levels (clamp). Similarly, hypopituitary patients with growth hormone deficiency are an ideal clinical situation to examine the potential role of growth hormone in regulating the systemic availability of FFA, thereby influencing the flexibility of ectopic lipids.

#### 3.1.1. IMCL

Studies on the effect of an acute bout of physical exercise on IMCL using ^1^H-MRS are summarized in Table 1. The decrease in IMCL during a single bout of exercise was present in almost all trials [37,38,43,44,45,46,47,48,49,50,51,52,53,54,55,65,66,67].

Most importantly, exercise protocols have to be designed in such a way that lipolysis is stimulated in order to induce a decrease in IMCL, meaning that exercise length and intensity need to be chosen accordingly. Differences in stimulation of lipolysis can impact on the changes in IMCL after exercise [56].

Quantitatively, stable isotope turnover studies suggest that up to 34% of the energy during exercise originates from non-free fatty acid oxidation in endurance-trained men and untrained humans [109,132], i.e., from IMCL and potentially also from VLDL since the stable isotope technique cannot distinguish between different sources of triglyceride oxidation.

The results are conflicting in exercise studies using skeletal muscle biopsies [81,82,85,110,111,133,134]. This may be due to methodological issues since biochemical analysis has a high between-biopsy variance [81]. Alternatively, different exercise protocols may account for the different findings.

The IMCL dynamics has been extensively investigated in healthy trained males [32,37,43,46,48,50,51,52,53,55,56,58,59,60,61,62,63,64,65,77,78,79,109,127]. In contrast, results on the effect of physical exercise on ectopic lipids in females are scarce [38,45,49,121,135,136,137]. The limited data, however, suggest that the capacity to deplete IMCL during prolonged exercise in sedentary subjects is higher in females than in males [121,135,136,137]. This might be due to the higher pre-exercise IMCL content in women [121,135,136,137,138]. It was shown that total body fat is highly correlated with IMCL content in sedentary subjects [139,140]. Since females are known to have higher total body fat, which could partly explain the higher pre-exercise IMCL in females. However, the results are not consistent in several studies [45,49,141]. With regard to the influence of gender on the flexibility of IMCL, Zehnder et al. showed a higher IMCL depletion in males than in females [49] with significantly higher pre-exercise IMCL levels in males than in females. Possibly different estrogen levels during the menstrual cycle and the corresponding effect on lipolysis may contribute to these findings [142]. The gender difference in skeletal muscle substrate metabolism on the molecular level is well known and is reviewed in [112].

Most of the data are based on healthy subjects. However, there are preliminary data in patients with growth hormone deficiency and type 1 diabetes, suggesting that IMCL do not behave differently in these clinical situations [65,66,67].

Mechanistically, during an acute bout of exercise the energy demand is increased. This energy is provided by glycolysis of glucose/glycogen or oxidation of fatty acids. The fatty acids are supplied either by intracellular lipolysis or by uptake of fatty acids from the blood stream. In the blood stream, triglycerides are transported within very low density lipoproteins (VLDLs) or chylomicrons and FFAs are bound to albumin. The triglycerides within the VLDLs or chylomicrons are hydrolyzed to FFA, a reaction catalyzed by the lipoprotein lipase. Lipoprotein lipase is mainly expressed in the endothelium of myocytes, cardiomyocytes and adipocytes [143,144]. The uptake of the corresponding FFAs to the skeletal or heart muscle are facilitated by specific FFA transporters (CD36, fatty acid transport protein, FABPpm) [145,146] but passive diffusion has also been reported [147].

The key enzymes involved in regulating lipolysis within the working tissues are the adipose triglyceride lipase [148] and the hormone sensitive lipase [149,150], which is inhibited by insulin [151] and—among others—stimulated by GH [129,130,131] and catecholamines [152]. Apart from the before-mentioned enzymes, other factors influence ectopic lipid degradation such as proteins coating the lipid droplets (e.g., perilipins), droplet size, and droplet localization [153].

While increased IMCL storage per se can be seen in healthy, insulin-sensitive athletes, it has also been shown that IMCL deposition in sedentary subjects can be associated with insulin resistance [154,155]. Samuel and Shulman showed an association of IMCL elevation, availability of lipotoxic intermediates, and insulin resistance [156]. It is currently unclear whether the increase in IMCL is just a consequence of insulin resistance or whether it plays an important role in the pathogenesis of mitochondrial dysfunction resulting in insulin resistance and type 2 diabetes mellitus [157]. Additionally, it is speculated that fatty acid metabolites such as diacylglycerol and ceramide play a more important role in inducing insulin resistance than triglyceride ectopic lipid deposition [140,158] per se. The possible mechanisms underlying the dynamics of IMCLs are reviewed in [159].

#### 3.1.2. IHCL

The clinical correlate of a pathological increase in IHCL is non-alcoholic fatty liver disease (NAFLD). NAFLD is associated with elevated mortality [160] and can evolve to an inflammation of the liver (NASH), fibrosis and then progress to cirrhosis and hepatocellular carcinoma [161]. Interestingly, type 2 diabetes is associated with a higher risk for hepatocellular carcinoma [162], which might be the consequence of a high prevalence of NAFLD in type 2 diabetes mellitus and insulin resistance [163] with an increased hepatic triglyceride synthesis in insulin resistant subjects [164]. Remarkably, elevated IHCL content is associated with hepatic insulin resistance and with peripheral insulin resistance as well [165] suggesting cross-talk between these two ectopic lipid deposits.

The few studies investigating the flexibility of IHCL following short-term exercise are summarized in Table 2.

Two studies reported a significant increase in IHCL immediately after an acute bout of physical exercise. These studies were performed in healthy trained subjects [43,44], and the results are consistent with other ^1^H-MRS studies [67,70]. However, these results are intriguing since energy expenditure is increased during exercise and NAFLD is mainly present in non-physically active overweight subjects [4]. It is established that during physical exercise systemic lipolysis increases, consistent with an increase in systemic FFA levels [166]. The increase in FFA concentrations is compatible with the fact that FFA availability during exercise increases and exceeds the required energy of the working tissues (i.e., skeletal muscles and the heart). Consequently, the excess of FFAs is transiently stored in the liver as IHCL [167], comparable to the concept of adipose tissue as a buffer for excessive lipid availability [168]. Similarly, Shulman described the ectopic lipid deposition as a consequence of a “spillover of energy storage from adipose tissue to the liver and skeletal muscle” [169]. The fact that an increase in IMCL was also observed in non-exercising muscle [50] corroborates this hypothesis.

Johnson et al. [70] confirmed the increase in IHCL, but documented this finding only 4.5 h after exercise, whereas Bilet et al. did not show significant changes of IHCL following an exercise of 2 h [69]. Since the latter study was performed in overweight subjects, it is conceivable that the background IHCL was higher in this population, resulting in only small relative differences of IHCL after exercise, not detected in this study. Additionally, whether differences in dietary preloading with fat before exercise impact on the changes in IHCL remains to be established [43].

Interestingly, an increase in IHCL after exercise was also observed in subjects with growth hormone deficiency. The increase was comparable to matched control subjects. Furthermore, growth hormone replacement therapy did not affect the flexibility of IHCL [67]. This indicates that the lipolytic action of growth hormone has a negligible effect on flexibility of ectopic lipids. It is conceivable that the redundant lipolytic hormone system including cortisol and catecholamines lead to a more than sufficient lipolysis and overcomes a single hormone deficiency.

#### 3.1.3. ICCL

ICCL accumulation was associated with impaired cardiac function [34,170] and appears to play a role in the development of diabetic cardiomyopathy, possibly mediated by lipotoxic intermediates [171].

Remarkably, Mantovani et al. recently showed that NAFLD might be associated with impaired cardiac function [172]. In addition, in non-diabetic subjects there is evidence that it is rather the increased IHCL than ICCL that correlates with diastolic dysfunction [173]. Interestingly, it is rather the pericardial fat than ICCL that correlates negatively with systolic function [174].

There are few studies investigating the effect of an acute bout of exercise on ICCL. After fat loading during three days, ICCL were significantly reduced after a two-hour bout of exercise, indicating that ICCL is also a flexible fuel store that can be used as an energy resource [43]. In contrast, Bilet et al. showed an increase in ICCL when fasting and exercising (2 h cycling) at 4 h after exercise while no significant change was seen when ingesting glucose during exercise with a tendency to decrease during exercise [175]. Glucose ingestion results in a release of insulin, which inhibits lipolysis and might, therefore, impact on ICCL consumption. Moreover, the diurnal variation of ICCL during a standardized day, which seems to be on the same order of magnitude as the changes induced by short-term exercise, could affect the results [101]. In conclusion, the evidence is limited and further studies are necessary.

### 3.2. Long-Term Effect: Physical Exercise

#### 3.2.1. IMCL

Several studies investigated the effect of long-term (1–6 months) physical exercise on IMCL. These studies were performed in healthy trained subjects, type 2 diabetic patients, and overweight subjects.

Consistently, healthy subjects showed an increase in the absolute amount of IMCL with training [39,62,76,85,113]. These findings are in line with the so-called “athlete’s paradox”. This term was used to describe the intriguing finding that IMCL levels in athletes were as high as those in obese, sedentary subjects or insulin-resistant subjects [114,115,116,117,118,119,120,121,158,176]. However, the capacity to deplete IMCL during exercise was increased in endurance-trained athletes, further corroborating the fact that IMCL can be considered as local fuel stores that are used during physical exercise in proportion to their pre-exercise content [37,45,47,56,59,62,111,135]. It is conceivable that increased IMCL related to training are beneficial for athletes since higher substrate stores are locally available during exercise, similarly to locally stored glycogen.

There are conflicting results in subjects with type 2 diabetes mellitus or impaired glucose tolerance showing an increase in IMCL with training [71], no absolute change [73] but changes in distribution within the muscle fibers with training [72], or a reduction of IMCL with training [74,75]. Various training intensities, different training session durations, or different diet protocols may lead to this inconsistency. Interestingly, insulin sensitivity consistently improved in type 2 diabetic patients as well as in obese non-diabetic subjects following long-term exercise [72,74,177], indicating that IMCL and the flexibility of IMCL are not the only factors that determine insulin sensitivity.

Morphological difference in lipid droplets’ distribution within the muscle fibers with higher subsarcolemmal lipids in insulin resistant subjects compared to highly trained subjects [72] indicate that different localization of lipid droplets within the myocyte may be critical for local lipid metabolism in trained athletes compared to insulin-resistant subjects.

#### 3.2.2. IHCL

Several trials investigated the amount of IHCL following exercise training for one to six months. Some studies reported a reduction in IHCL after training intervention in healthy subjects [123,124,178,179,180] or type 2 diabetes patients [73]. In subjects with NAFLD, the results were conflicting with a significant decrease in IHCL following 8–16 weeks of endurance [122,181], high-intensity interval [182], or resistance training [183], but only a tendency to reduce IHCL after 16 weeks of aerobic exercise [125]. Remarkably, reduction in IHCL was accompanied by a higher skeletal muscle and adipose tissue insulin sensitivity but without any change in hepatic insulin sensitivity [181].

#### 3.2.3. ICCL

Data on long-term interventions investigating ICCL are scarce. In type 2 diabetes, a six-month exercise intervention resulted in a reduction in paracardial fat in parallel with a reduction in visceral fat. The amount of ICCL, however, did not significantly change [73]. These findings are in agreement with another trial investigating ICCL content before and 12 weeks after a training program in overweight patients with type 2 diabetes [177]. In contrast, in obese subjects without type 2 diabetes mellitus a 12-week training intervention decreased ICCL significantly in parallel with an improved ejection fraction [126].

The reduction in paracardial fat was most likely related to the loss of whole body adipose tissue. The lack of significant changes in ICCL may be due to the fact that the heart muscle depends mainly on lipids as energy sources at baseline and during exercise [184,185]. Data on the flexibility of ICCL are limited and more studies are needed to confirm these findings.

## 4. Nutritional Interventions

### 4.1. IMCL

Studies on the effect of short-term nutritional interventions on IMCL are summarized in Table 3. Nutritional intervention studies have mainly been performed in healthy individuals. The fasting condition is associated with low insulin levels resulting in a disinhibition of lipolysis leading to an increase in fat availability as documented by an increase in FFA concentrations. This effect can even be augmented by the effect of insulin antagonists such as catecholamines, cortisol, GH, and glucagon. It is, therefore, not surprising that a fasting period of 2–5 days increased IMCL [23,24,25,26]. In contrast, a short duration fasting period (12 h) resulted in a decrease in IMCL; unfortunately the underlying mechanism remains unclear since information on other metabolic parameter such as FFA availability is lacking in this study [27].

When combining the effect of fasting and exercise, both inducing lipolysis, the effect on IMCL consumption during exercise is additive, meaning that IMCL breakdown during exercise in exercising skeletal muscle is increased in the fasted state [58].

On the other hand, standardized increased lipid availability in the presence of hyperinsulinemia can be induced either by an intravenous infusion of FFA paralleled by a hyperinsulinemic–euglycemic clamp or a high-fat diet with co-ingestion of carbohydrates (CHO). In either situation hyerinsulinemia inhibits systemic and local lipolysis.

A high-fat diet for 2–3 days resulted in a significant increase in IMCL [28,29,30,31] as well as a high-fat diet for six weeks [32], whereas a single high-fat meal did not increase IMCL in lean subjects [33]. These conflicting data may be related to the amount of fat that is available to replete IMCL. A single high-fat meal results in lower fat availability compared to repetitive high-fat meals [189].

Consistently, intravenous lipid infusion of long-chain fatty acids (soybean oil) during a hyperinsulinemic–euglycemic clamp induced an increase in IMCL [28,186,187,188] suggesting that insulin has an important role in facilitating the repletion of IMCL. Interestingly, lipid infusion alone increased IMCL in healthy volunteers in one study [190] whereas a mixture of medium and long chain FFA did not increase IMCL in two different studies [28,40]. Importantly, with or without increase in IMCL, infusion of FFA, independent of the FFA chain length, leads to peripheral insulin resistance [187], indicating that the increase in IMCL is not the only factor that is related to peripheral insulin resistance.

Importantly, starvation (67 h) and a high-fat diet had a comparable effect on IMCL [41], probably because systemic fat availability increased in both situations.

After physical exercise, a high-fat diet replenished IMCL [24,37,38,39,42,191,192]. High- and low-fat diets repleted IMCLs differently after an acute bout of exercise [42], indicating that dietary fat availability following exercise is critical in repleting IMCL. However, training status (i.e., sedentary vs. endurance-trained subjects) did not significantly affect the speed of repletion [39]. Similarly, repletion of IMCL was observed in the situation of a post-exercise fasting period [54]. In this condition, the increased fat availability is related to an increase in systemic lipolysis, mainly from adipose tissue, as evidenced by an increase in FFA concentrations. A high-fat diet administered over 2.5 days before a short bout of exercise resulted in a higher pre-exercise IMCL content but also a higher reduction in IMCL [37] following exercise, indicating that local lipid fuel stores are preferentially used in case of physical exercise. Similar results were seen after low or high systemic CHO availability during exercise with a higher (in case of low CHO availability) or lower IMCL (high CHO availability) depletion, indicating that primarily CHO are used as fuel, if available [52]. This is consistent with the observation that IMCL decreased during exercise in fasting subjects but not in subjects ingesting CHO [58]. Most likely, these findings were mediated by the higher insulin levels during the CHO-rich diet, resulting in decreased lipolysis during exercise.

A calorie-restricted diet resulting in weight loss reduced overall IMCL significantly [116,193,194,195]. Also, an isocaloric, very low-fat diet reduced intramuscular triglyceride concentration [196]. However, when weight loss was combined with exercise training, pre-exercise IMCLs increased in the exercising muscles [116], indicating that the training effect exerts a more prominent effect on IMCL than weight reduction.

In overweight men, a high-fat diet for three weeks did not lead to IMCL accumulation [197]—in contrast to lean sedentary subjects or athletes, where a high-fat diet for 1–7 weeks increased IMCL content [191,192,198,199,200,201].

Data in patients with type 1 diabetes and hypopituitarism suggest that the flexibility of IMCL after dietary intervention is not significantly different from healthy matched control subjects [65,66]. However, typical insulin-resistant subjects may have decreased flexibility of IMCL [111].

In summary, short-term high fat availability induced by starvation, lipid infusion, or dietary fat intake increases IMCL, in particular in the presence of hyperinsulinemia. In contrast, long-term caloric restriction tends to reduce IMCL. The present evidence again suggests that IMCL are flexible fuel stores. However, the flexibility of IMCL is not related to insulin resistance alone, but is regulated by a complex interplay including diet, fat availability, physical exercise, and insulin action.

### 4.2. IHCL

Data on flexibility of IHCL following dietary intervention are scarce and controversial. Studies on the effect of short-term nutritional interventions on IHCL are summarized in Table 4. A short-term very low calorie diet for three days resulted in a decrease in IHCL in men [34]; however, a fasting period of 48 h resulted in a significant increase in IHCL in men, but not in women [23].

Remarkably, the available data suggest that a high-fat meal resulted in an increase in IHCL in men. Unfortunately, data on the effect on a high-fat meal on IHCL in women are lacking [33,35,36]. In contrast, compared to a mixed (isocaloric) diet, a high-fat diet did not influence IHCL before a physical exercise intervention but both interventions led to an increase in IHCL after exercise [70], indicating that exercise probably exerts a more prominent effect on IHCL than a short-term diet intervention.

The long-term effect of nutrition on IHCL is mainly studied with a calorie-restricted diet for up to 16 weeks, resulting in a reduction in IHCL in healthy subjects [202,203,204] as well as in patients with type 2 diabetes mellitus or non-alcoholic hepatic steatosis [205,206,207].

In contrast, an iso- or hypercaloric high-fat diet for one to six weeks induced an IHCL accumulation in healthy normal weight [208] and overweight men [197], as well as in obese women [209]. A high-fat diet with polyunsaturated fatty acids did not affect IHCL deposition, while saturated fatty acids increased liver fat significantly [210].

However, a high-fat (59%–75% fat) hypocaloric diet for two weeks to six months resulted in a decrease in IHCL [211,212,213]. Surprisingly, an increase in IHCL after a high-fat diet for four days was not accompanied by an alteration in insulin sensitivity [36]. Similarly, a high-fat diet for three weeks did not affect insulin sensitivity in healthy overweight men [36,197], despite an increase in IHCL. However, in lean subjects, a high-fat diet resulted in reduced hepatic insulin sensitivity [214]. Although—in general—the amount of IHCL is positively correlated with insulin resistance (in contrast to IMCL), the relation between IHCL, the flexibility of IHCL, and insulin action is probably more complex than previously thought and more studies are needed to understand the underlying mechanisms.

Sucrose-sweetened beverages increased IHCL in overweight non-diabetic subjects and healthy subjects [215]. This is consistent with the observation that subjects with NAFLD consumed more soft drinks than healthy controls (comparable daily CHO intake) with a positive correlation of severity of NAFLD and amount of consumed soft drinks [216]. In general, a high-fructose diet led to an IHCL accumulation [217,218,219,220], while a reduction of consumption of sugar-sweetened beverages led to a substantial reduction in IHCL in obese subjects [221]. The increased de novo lipogenesis after a high-fructose or -glucose diet could contribute to this finding [222,223,224,225] as well as the antilipolytic effect of insulin, which is secreted after ingestion of glucose-containing drinks. The effect of fructose on IHCL was dose-dependent since a lower amount of fructose over four weeks did not affect IHCL content [226].

The effect of a high-glucose diet on IHCL was comparable to that of a high-fructose diet [217,219,227]. Adding protein to a single meal did not blunt IHCL accumulation [33]. In contrast, when adding proteins or amino acids to a high-fat or high-fructose diet for 4–6 days, IHCL accumulation was lower without affecting insulin sensitivity [36,220]. When adding protein to an equilibrated diet, IHCL were significantly lowered in obese subjects [228], again without change in glucose tolerance. The underlying mechanism is unclear.

In summary, a short-term increase in fat availability by starvation, exercise, or dietary fat increased IHCL, whereas long-term starvation tended to decrease IHCL. Similar to IMCL, the current data suggest that IHCL are flexible fuel stores. IHCL are significantly related to insulin resistance, but the regulation of IHCL is a complex interplay between quantitative and qualitative diet (i.e., fat, fructose, protein), insulin action, and probably physical exercise.

### 4.3. ICCL

A high-fat diet did not affect ICCL content [35,102], even in the presence of increased serum triglyceride levels [102]. Moreover, cardiac function was not affected [35]. However, a 48-hour fasting period resulted in a significant increase in ICCL in healthy men [102]. Similarly, a short-term low-calorie diet induced a significant increase in ICCL in healthy men [34], as well as in subjects with type 2 diabetes mellitus [229]. This was likely due to the increased FFA levels during starvation or a low-calorie diet.

In contrast, a prolonged hypocaloric diet decreased ICCL [206] and improved myocardial function. However, the very low calorie diet also resulted in significantly lower blood pressure and body weight. Both of them may have a beneficial impact on myocardial function.

### 4.4. Effect of Bariatric Surgery on Ectopic Lipids

The most effective treatment on the cardiometabolic risk profile is bariatric surgery. Data on the effect of bariatric surgery on ectopic lipids are scare. The current evidence suggests that in addition to visceral fat mass loss, IHCL mainly decrease after six months, whereas ICCL remain unchanged initially but tend to decrease after a longer observation period (>32 months) [230].

## 5. Genetics and Drugs

### 5.1. Genetic Background of Ectopic Lipids

Beyond diet and exercise, genetic disorders—the so-called lipodystrophy syndromes—can impact on lipid storage within non-adipose tissue. Lipodystrophies are a rare and heterogenous group of disorders, characterized by a complete or a partial lack of white adipose tissue [231]. In general, the amount of fat loss correlates with the associated metabolic abnormalities, including severe insulin resistance (acanthosis nigricans), hypertriglyceridemia, and an increase in ectopic lipid storages, in particular in the liver, which, in turn can lead to inflammation or nonalcoholic steatohepatitis (NASH), fibrosis, and finally hepatocellular carcinoma [232]. It would go beyond the scope of the current review to summarize the understanding of the known lipodystrophy syndromes and their underlying genetic mechanisms (see also [233]). Briefly, mesenchymal stem cells have the capacity to differentiate into adipocytes. They differentiate, firstly, into pre-adipocytes, then into adipocytes (stimulated by insulin, glucocorticoids, IGF-1, and prostaglandines), which in turn differentiate into a mature adipocyte before undergoing apoptosis [234]. Along this pathway several regulatory factors can be mutated, leading to a loss of fat tissue, thereby impairing the lipid handling and storage in the adipose tissue [233]. This results in an excess of lipids in non-adipose tissues. However, whether ectopic lipid accumulation is a sign of disease or a physiological response in patients who have little capacity to store lipids is not clear. Importantly, the major difference between the lipodystrophy syndromes and their associated metabolic abnormality and obesity is the decrease in adipose tissue with low levels of leptin in lipodystrophy, whereas obesity is characterized by an excess of adipose tissue and increased leptin levels [235]. These observations underscore the importance of adipocyte biology in humans.

Lipodystrophy can be considered an extreme version of the obesity-associated metabolic features, including ectopic fat accumulation. It is, therefore, likely that diet and exercise do not influence the flexibility of ectopic lipid stores in a very significant way, but currently no data are available. In contrast, leptin replacement therapy has been shown to result in a significant improvement of the metabolic abnormalities associated with lipodystrophy (including a decrease in IMCL and IHCL) [236,237,238]. Leptin has, therefore, been approved in Japan and USA for the treatment of diabetes and hypertriglyceridemia in patients with lipodystrophy.

### 5.2. Medical Therapy for Ectopic Lipids

Besides diet and exercise, medical therapy has been investigated in the context of ectopic lipids. The main focus of medical investigations have been IHCL since non-alcoholic steatohepatitis (NASH) is now the most common cause of liver disease and may in the future be the main reason for liver transplantation [239]. Currently there is no approved drug therapy for NASH, but there are encouraging results in phase II trials. In the largest randomized-controlled trials in patients with NASH, treatment with pioglitazone, vitamin E [240], and obeticholic acid [241] were associated with improvements in liver histology and/or IHCL relative to placebo. However, long-term safety concerns remain, especially for pioglitazone and vitamin E administration. Recently, the effect of GLP-1 analogues on NASH was tested in two randomized-controlled trials. Using ^1^H-MRS the GLP-1 analogue exenatide has been shown to significantly decrease IHCL and epicardial fat, whereas IMCL and intrapancreatic lipids remained unchanged [242]. Similarly, liraglutide therapy, a long-acting GLP-1 analogue, resulted in significant histological improvement of NASH after 48 weeks of therapy [243]. Both compounds were safe and larger phase III studies are awaited.

## 6. Conclusions

Ectopic lipids such as IMCL, IHCL, and ICCL are metabolically active fuel stores. An acute bout of exercise depletes ectopic lipids in “working tissues” (i.e., skeletal muscle and heart muscle) and increases them in the liver. Short-term high-fat dietary intervention leads to repletion of IMCL, whereas the effect of short-term dietary intervention on IHCL is less clear and probably depends on quantitative and qualitative content of the diet. In contrast, a high-fat diet does not seem to affect ICCL. However, we have to acknowledge that there are very limited data available on the effect of a high-fat diet on ICCL.

Short-term starvation results in an increase in IMCL in non-working muscles, in IHCL, and ICCL, whereas long-term caloric restriction tends to decrease IMCL, IHCL, and ICCL. The current evidence suggests that in particular the increased flexibility of IMCL is related to training status and a hallmark of endurance trained athletes, further corroborating the fact that IMCL are metabolically active local fuel stores.

In addition to diet and physical exercise, insulin action plays an important role in regulating the flexibility of ectopic lipids. However, the exact underlying mechanisms are not fully established. Interestingly, preliminary data suggest that the flexibility of ectopic lipids is not only observed in healthy subjects but also in patients with a lack of hormones involved in lipid metabolisms such as growth hormone deficiency and type 1 diabetes mellitus.

Congenital lipodystrophies are rare and heterogeneous genetic disorders characterized by a complete or partial lack of subcutaneous tissue resulting in an overflow of fat metabolites in ectopic tissues. Metabolically, they can be considered an extreme version of the obesity-associated metabolic features. In contrast to obesity-related metabolic abnormalities, lipodystrophy is associated with a lack of leptin and leptin replacement therapy has been shown to improve insulin resistance and hypertriglyceridemia in patients with lipodystrophy. The main focus of medical investigations has been IHCL. Encouraging results of randomized controlled phase II trial have been reported with pioglitazone, vitamin E, obeticholic acid, and GLP-1 agonists. However, so far there is no drug approved for the therapy of steatohepatitis.

**Figure 4:**
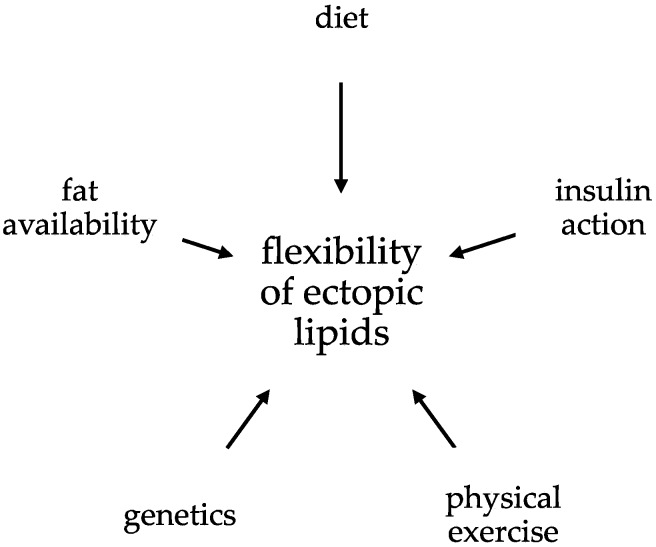
Factors influencing lipids: See text for details.

The available evidence suggests that a complex interplay including quantitative and qualitative diet, fat availability (fat mass, FFAs), insulin action, genetic background [244], and physical exercise are important factors that influence ectopic lipids (Figure 4). Furthermore, the time frame of the intervention on these parameters (short-term vs. long-term) appears to be critical. Consequently, standardization of physical activity and diet is mandatory when assessing ectopic lipids in predefined clinical situations.

## Figures and Tables

**Table 1:** Effect of short-term exercise on IMCL using ^1^H-MR-Spectroscopy.

Author (Year)	*n*	Subjects	Gender	Intervention	IMCL	% Change	Muscle Investigated	Comments
Christ (2016) [67]	10	Volunteers with adult-onset GHD	m, f	2 h exercise at 50%–60% VO_2_ max on a treadmill	↓ *	−9.3 to −13.5	M. tibialis anterior	No significant effect of growth hormone replacement therapy on IMCL and IHCL, IHCL ↑ *
Bucher (2014) [43]	10	Healthy volunteers	m	2 h exercise on bicycle ergometer at 50%–60% VO_2_ max	↓ *	−16.8	M. vastus intermedius	IHCL ↑ *, ICCL ↓ *
Egger (2013) [44]	18	Healthy volunteers	m, f	2 h exercise on treadmill at 50%–60% VO_2_ max	↓ *	−22.6	M. tibialis anterior	IHCL ↑ *
Vermathen (2012) [47]	8	Trained cyclists or runners	m	3 h exercise on bicycle ergometer or treadmill at 50% W_max_	↓ *	−3 to −50	Thigh (M. vastus intermedius, vastus lateralis, vastus lateralis, adductor magnus, biceps femoris; rectus femoris) or lower leg muscle (tibialis anterior, soleus lateralis, soleus medialis, gastrocnemius lateralis, gastrocnemius medialis, extensor digitorum)	In M. biceps femoris and rectus femoris no significant decrease
Jenni (2008) [65]	7	Physically active men with T1DM	m	2 h cycling at 55%–60% VO_2_ max	↓ *	−11.5 to −16.2	M. vastus intermedius	
Trepp (2008) [66]	15	Volunteers with adult-onset GHD	m, f	1 h walking at heart rate corresponding to 50% VO_2_ max, on three days and low fat diet	↓ *	−35 to −47.5 **	M. tibialis anterior	No significant effect of growth hormone replacement therapy on IMCL
De Bock (2007) [48]	9	Physically active men	m	2 h cycling at 75% VO_2_ peak	↓ *	−47	M. vastus lateralis	
Zehnder (2006) [37]	11	Endurance trained cyclists	m	3 h cycling at 50% W_max_	↓ *	−21 to −41	M. vastus intermedius	
Zehnder (2005) [49]	18	Cyclists or triathletes	m, f	3 h cycling at 50% W_max_	↓ *	−42 to −59	M. vastus intermedius	Larger reduction in males
Schrauwen-Hinderling (2003) [50]	8	Highly trained cyclists	m	3 h cycling at 55% W_max_	↓ *	−20.4	M. vastus lateralis	M. biceps brachii ↑ *
Van Loon (2003) [51]	9	Endurance-trained cyclists	m	3 h cycling at 55% W_max_	↓ *	−21	M. vastus lateralis	No difference between normal and low-fat diet
White (2003) [46]	9	Moderately active	m	45 min cycling, intervals at 50% and 110% of ventilator threshold	↓ *	−38	M. vastus lateralis	
White (2003) [45]	18	Moderately active	m, f	1 h cycling at 65% VO_2_ max	↓ *	−11.5 to −17.1	M. vastus lateralis	
Johnson (2003) [52]	6	Highly trained cyclists	m	3 h cycling at 70% VO_2_ max	↓ *	−57 to −64	M. vastus lateralis	Higher IMCL degradation in low carbohydrate condition
Larson-Meyer (2002) [38]	7	Well-trained endurance runners	f	2 h running at 65% VO_2_ max	↓ *	−25	M. soleus	
Brechtel (2001) [53]	12	Well-trained subjects	m	Running: parallel design 60%–70% VO_2_ max, 80%–90% VO_2_ max 21/42 km	↓	−10 to −42	M. tibialis anterior, M. soleus	
Krssak (2000) [54]	9	Trained subjects	m, f	3–4 bouts of 45 min of running at 65%–70% peak oxygen until exhaustion	↓ *	−33.5 **	M. soleus	
Rico-Sanz (2000) [55]	5	Trained subjects	m	90 min running at 64% VO_2_ max	↓ *	−15.7 to −32.2 **	M. soleus, tibialis, gastrocnemius	in M. gastrocnemius no sign decrease
Rico-Sanz (1998) [68]	8	Trained subjects	m	13.2 km running, jogging, sprinting	→	+9 to −2.4 **	M. soleus, gastrocnemius, tibialis	

*n*: number of subjects; IMCL: intramyocellular lipids comparison pre- and post-exercise; *: significant (*p* < 0.05); IHCL: intrahepatocellular lipids; ICCL: intracardiomyocellular lipids; MRS: ^1^H-MR-Spectroscopy; T1DM: Type 1 diabetes mellitus; m: male; f: female; GHD: growth hormone deficiency; % change: relative change from baseline (in percentage); **: original values converted to relative change; ↓: decrease; ↑: increase; →: no change.

**Table 2:** Effect of short-term exercise on IHCL using ^1^H-MR-Spectroscopy.

Author (Year)	*n*	Subjects	Gender	Intervention	IHCL	Comments
Christ (2016) [67]	10	Volunteers with adult-onset GHD	m, f	2 h exercise at 50%–60% VO_2_ max on a treadmill	↑ *	No significant effect of growth hormone replacement therapy on IMCL and IHCL, IMCL ↓ *
Bilet (2015) [69]	21	Overweight subjects	m	2 h cycling at 50% W_max_	→	
Bucher (2014) [43]	10	Healthy volunteers	m	2 h cycling at 50%–60% VO_2_ max	↑ *	ICCL ↓ *, IMCL ↓ *
Egger (2013) [44]	18	Healthy volunteers	m, f	2 h aerobic exercise on treadmill at 50%–60% VO_2_ max	↑ *	
Johnson (2012) [70]	6	Healthy trained volunteers	m	90 min cycling at 65% VO_2_ peak	↑ *	At 4.5 h post-exercise

*n*: number of subjects; IHCL: intrahepatocellular lipids comparison pre- and postexercise; *: significant (*p* < 0.05); IMCL: intramyocellular lipids; ICCL: intracardiomyocellular lipids; MRS: ^1^H-MR-Spectroscopy; T1DM: Type 1 diabetes mellitus; m: male; f: female; GHD: growth hormone deficiency; ↓: decrease; ↑: increase; →: no change.

**Table 3:** Effect of short-term dietary interventions on IMCL.

Author (Year)	*n*	Subjects	Gender	Intervention	IMCL	Comments: Method, Muscle Investigated
Browning (2012) [23]	18	Healthy individuals	m, f	Fasting for 48 h	↑ *	^1^H-MRS M. soleus, only in women, not in men
Green (2010) [24]	6	Healthy physically fit men	m	Fasting for 67 h	↑ *	^1^H-MRS M. vastus lateralis
Stannard (2002) [25]	6	Nondiabetic, physically fit men	m	Fasting for 72 h	↑ *	^1^H-MRS M. vastus lateralis
Wietek (2004) [26]	4	Healthy volunteers	m, f	Fasting for 120 h	↑ *	^1^H-MRS M. tibialis anterior, soleus
Machann (2011) [27]	12	Healthy volunteers	m	Fasting for 12 h	↓ *	^1^H-MRS M. tibialis anterior, soleus
Bachmann (2001) [28]	12	Healthy volunteers	m	High-fat diet for 3 days	↑ *	^1^H-MRS M. tibialis anterior, soleus (increase in M. tibialis, not in M. soleus)
Sakurai (2011) [29]	37	Healthy volunteers	m	Isocaloric, high-fat diet for 3 days	↑ *	^1^H-MRS M. tibialis anterior, M. soleus
Zderic (2004) [30]	6	Endurance-trained cyclists	m	Isocaloric, high-fat diet for 2 days	↑ *	Biopsy M. vastus lateralis
Larson-Meyer (2008) [31]	21	Endurance-trained runners	m, f	Isoenergetic, high-fat diet for 3 days	↑ *	Biopsy M. vastus lateralis Sign higher
Lindeboom (2015) [33]	9	Lean healthy subjects	m, f	Single high-energy, high-fat meal	→	^1^H-MRS M. tibialis anterior, ↑ * IHCL
Brechtel (2001) [186]	5	Healthy male subjects	m	5 h hyperinsulinemic euglycemic clamp and intralipid infusion	↑ *	^1^H-MRS M. tibialis anterior, M. soleus
Bachmann (2001) [28]	12	Healthy volunteers	m	6 h lipid infusion during hyperinsulinemic euglycemic clamp	↑ *	^1^H-MRS M. tibialis anterior, M.soleus; only in presence of insulin infusion
Hoeks (2012) [187]	9	Healthy lean males	m	6 h euglycemic hyperinsulinemic clamp and lipid or glycerol infusion	↑ *	Only in long-chain triacylglycerols emulsion, not in medium chain glycerols emulsion Biopsy M. vastus lateralis
Lee (2013) [188]	28	Normal-weight adolescents	m,f	12 h lipid infusion and 3 h hyperinsulinemic euglycemic clamp	↑ *	^1^H-MRS M. tibialis anterior
Brehm (2010) [40]	8	Glucose-tolerant volunteers	m	3 h Euglycemic pancreatic clamp, and intralipid infusion	→	^1^H-MRS M. soleus

*n*: number of subjects; IMCL: intramyocellular lipids comparison pre- and post-intervention or control diet; *: significant (*p* < 0.05); IHCL: intrahepatocellular lipids; ^1^H-MRS: ^1^H-MR-Spectroscopy; ↓: decrease; ↑: increase; →: no change.

**Table 4:** Effect of short-term dietary interventions on IHCL using ^1^H-MR-Spectroscopy.

Author (Year)	*n*	Subjects	Gender	Intervention	IHCL	Comments
Van der Meer (2007) [34]	14	Healthy, non-obese men	m	3 days very low calorie diet	↓ *	ICCL increased
Browning (2012) [23]	18	Healthy individuals	m, f	48 h fasting	↑ *	in males, no sign increase in women
Lindeboom (2015) [33]	9	Lean healthy subjects	m, f	Single high-energy, high-fat meal	↑ *	
Van der Meer (2008) [35]	15	Healthy men	m	3 days high-fat, high-energy diet	↑ *	No effect on ICCL
Bortolotti (2009) [36]	10	Healthy young men	m	4 days hypercaloric high-fat diet	↑ *	Protein co-ingestion blunts effect of high fat diet
Johnson (2012) [70]	6	Healthy trained males	m	High-fat diet	→	compared to Isocaloric control diet
Kirk (2009) [211]	22	Obese subjects	m, f	48 h energy-deficient, high-fat diet	↓ *	
Ngo Sock (2010) [217]	11	Healthy men	m	7 days hypercaloric, high-fructose diet	↑ *	
Lê (2009) [218]	24	Healthy offspring of T2DM patients and control subjects	m	7 days high-fructose diet	↑ *	also significant increase in IMCL
Lecoultre (2013) [219]	55	Healthy young males	m	6–7 days high-fructose diet	↑ *	Only if at least 3 g fructose/kg/day
Theytaz (2012) [220]	9	Healthy male volunteers	m	6 days high-fructose diet	↑ *	Supplementation with amino acids blunts increase

*n*: number of subjects; IHCL: intrahepatocellular lipids: comparison of pre- vs. post-intervention or control diet; *: significant (*p* < 0.05); IMCL: intramyocellular lipids; ICCL: intracardiomyocellular lipids; m: male; f: female; T2DM: type 2 diabetes mellitus; ↓: decrease; ↑: increase; →: no change.
